# Supplementary figures and images for: Evolutionary innovation through transcription factor rewiring in microbes is shaped by levels of transcription factor activity, expression, and existing connectivity
Source: PLoS Biol. 2023 Oct 23;21(10):e3002348. doi: 10.1371/journal.pbio.3002348 (PMC10621929; doi:10.1371/journal.pbio.3002348)

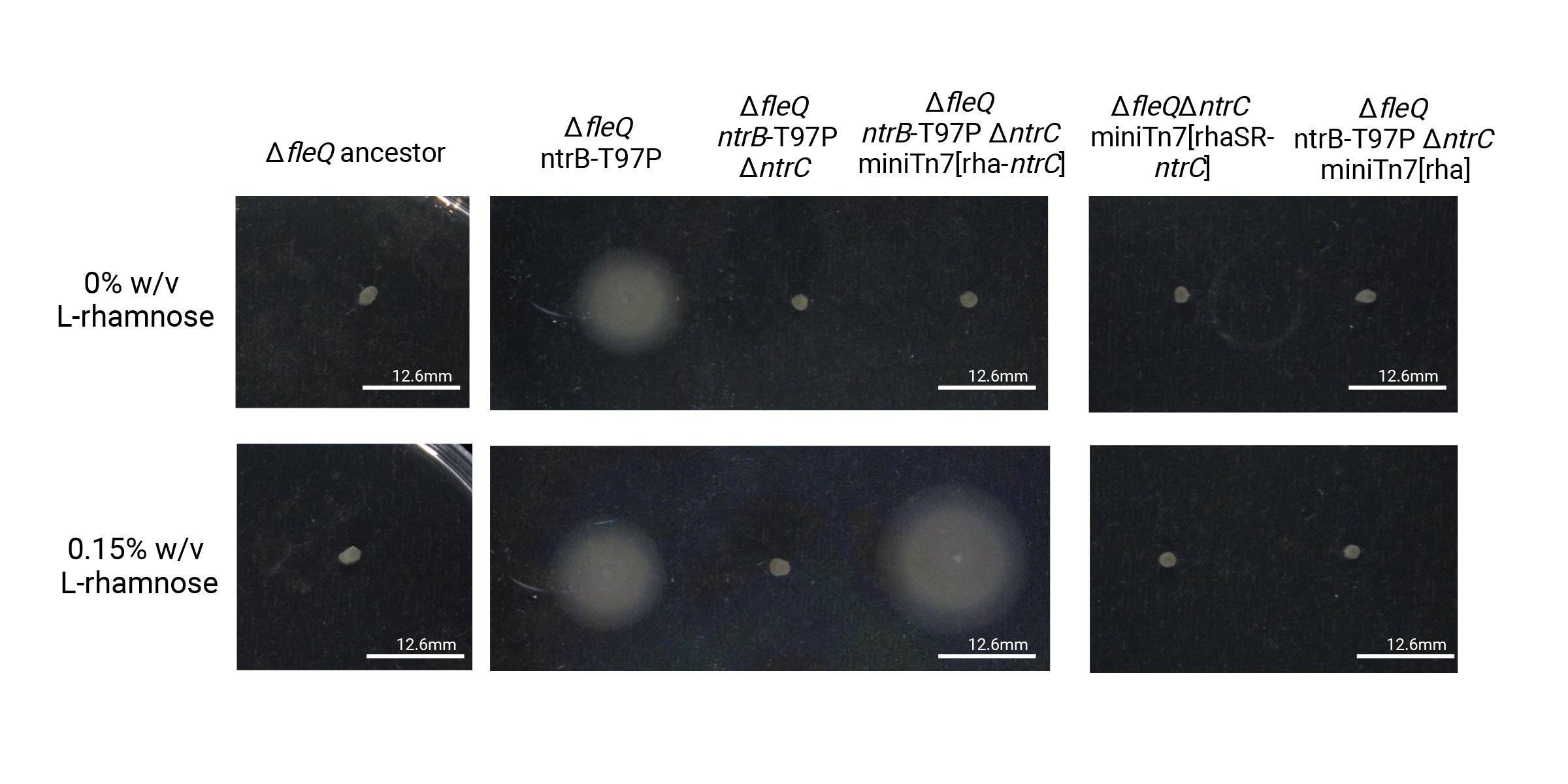

Supplement: S1 Fig — Transcription factor gene ntrC was deleted and then reintroduced as a single-copy chromosomal insertion expressed from an L-rhamnose–inducible promoter system (rhaSR-PrhaBAD). The same complementation lacking the ntrB-T97P mutation as well as an empty expression system transposon were included as further controls. Photographs of motility after 1-day incubation in 0.25% agar LB plates supplemented with or without 0.15% L-rhamnose for induction of transcription factor expression. (TIF) [file pbio.3002348.s001.tif]

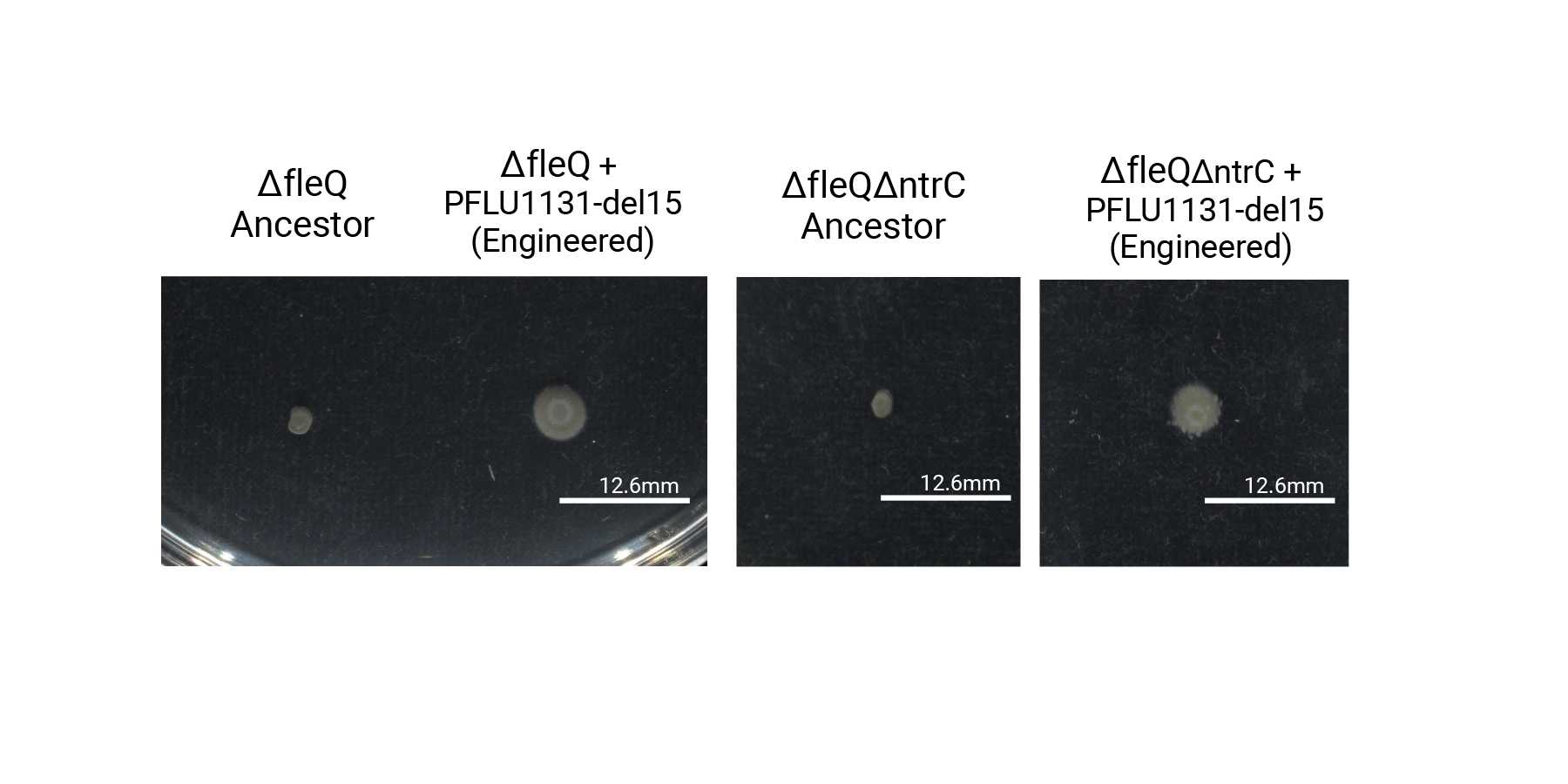

Supplement: S2 Fig — This indicates that rescue of motility by this mutation does not depend on ΔntrC and is the sole mutation required to do so. (TIF) [file pbio.3002348.s002.tif]

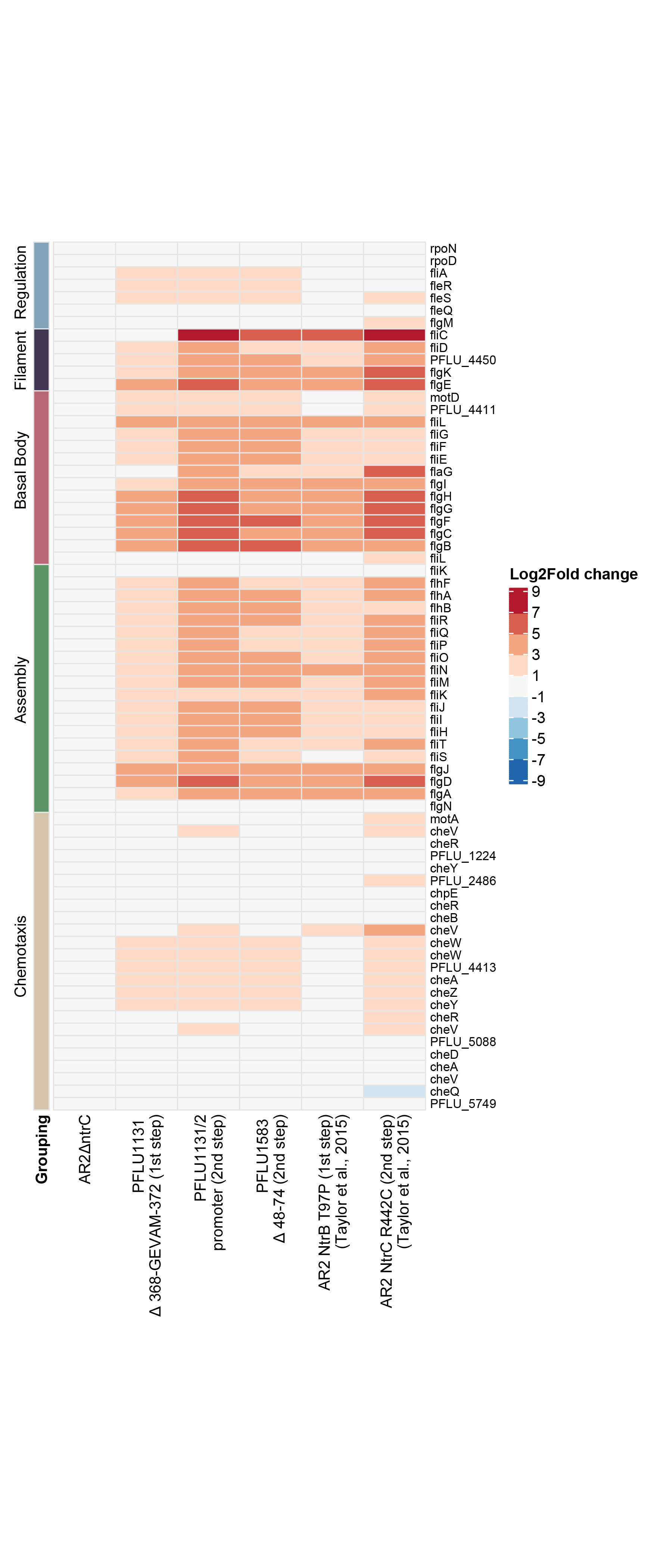

Supplement: S3 Fig — Log2Fold changes in gene expression relative to the ΔfleQ ancestor are shown for all genes associated with flagellar motility in P. fluorescens SBW25. Functional groups of genes are indicated by the coloured bars and labels to the left of the plot. Data underlying this figure can be found in S15 File. (TIF) [file pbio.3002348.s003.tif]

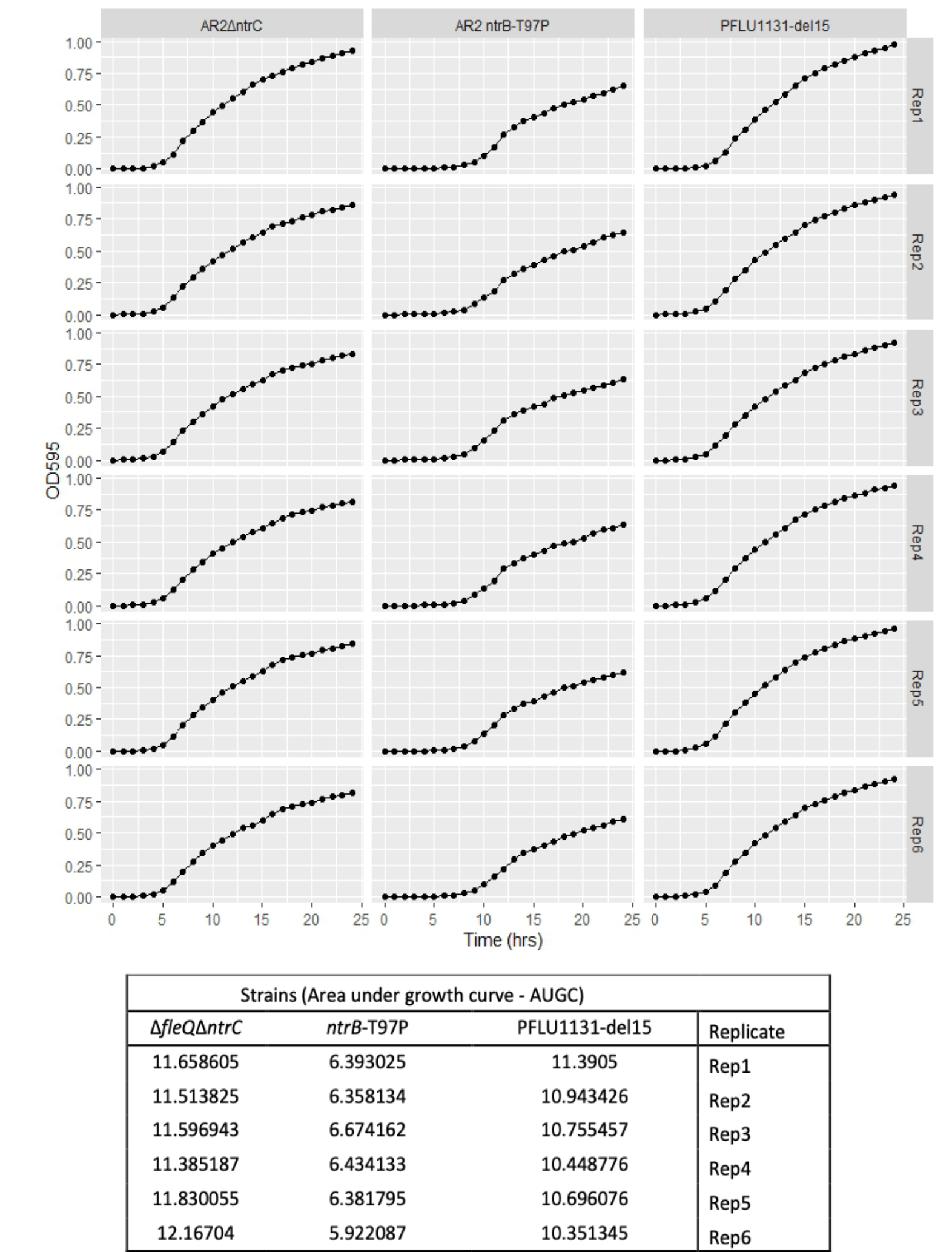

Supplement: S4 Fig — Six biological replicates for each of the 3 strains were assayed for change in OD595 over 24 hours of incubation in shaking LB. AUGC values corresponding to each curve are displayed in the table below the graphs. Data underlying this figure can be found in S10 File. (TIF) [file pbio.3002348.s004.tif]

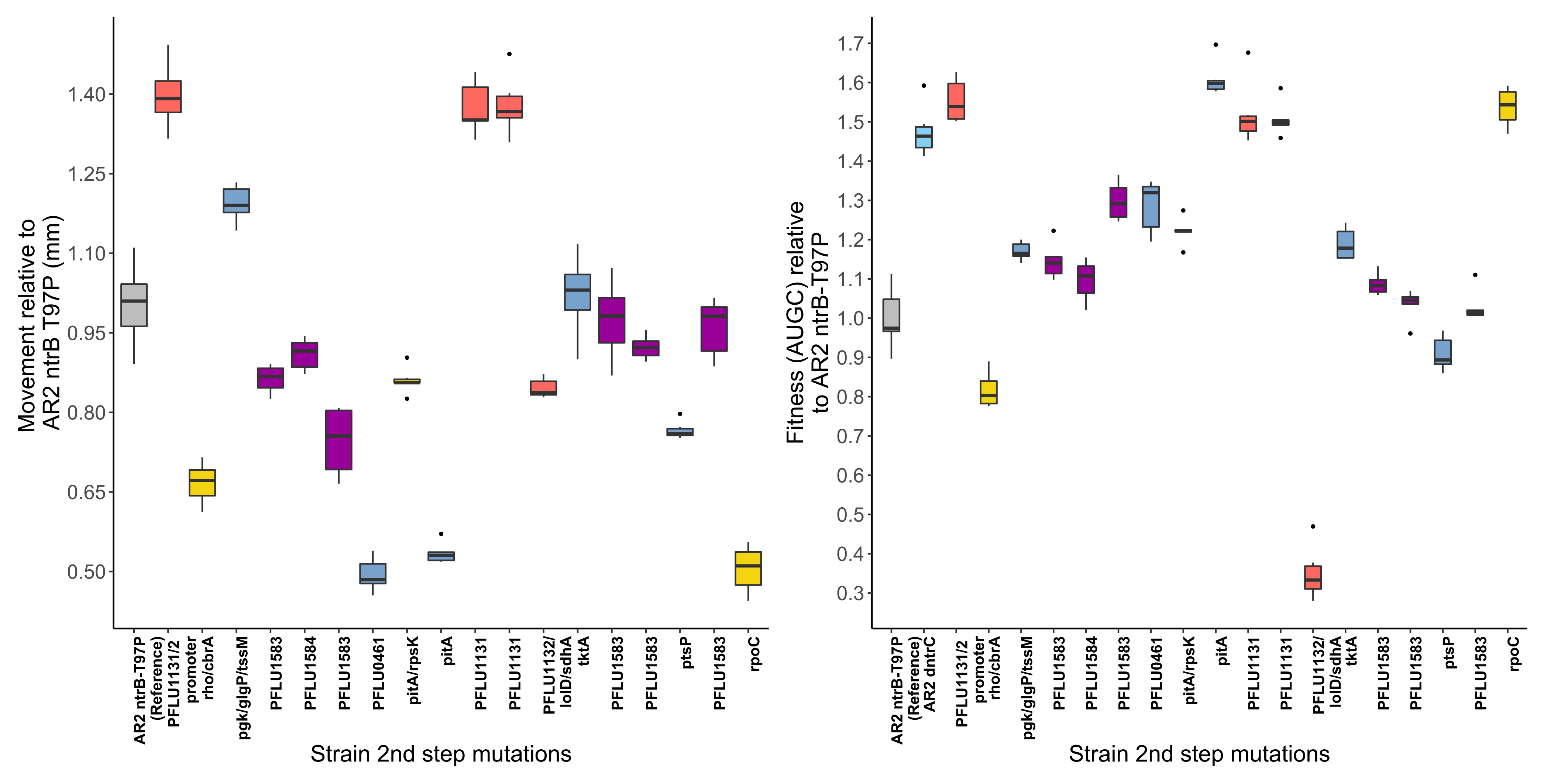

Supplement: S5 Fig — (A) Race assay as measure of motility fitness. Distance moved over 24 hours in 0.25% agar LB plates measured relative to the ΔfleQ ancestor ntrB-T97P mutant. (B) Fitness in LB measured as area under the 24-hour growth curve (AUGC) relative to ΔfleQ ancestor ntrB-T97P. In both plots, mutations are coloured by functional category: red: PFLU1131/2; purple: PFLU1583/4; blue: metabolic; yellow: global regulatory; grey: other. For all boxplots: box represents first to third quartile range, middle line represents median value, whiskers range from quartiles to maxima and minima. Data underlying parts A and B figure can be found in S16 and S17 Files, respectively. (TIF) [file pbio.3002348.s005.tif]

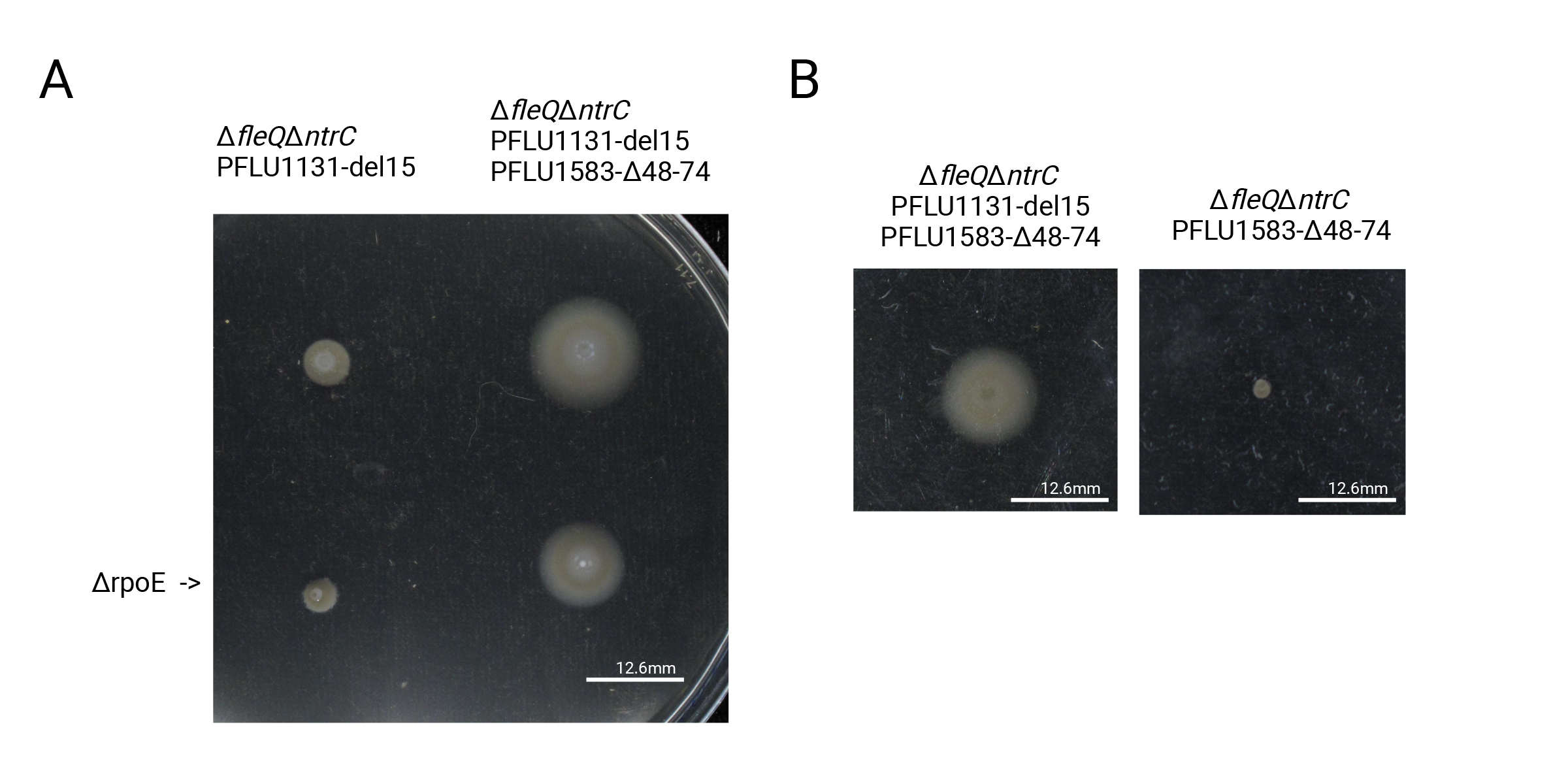

Supplement: S6 Fig — (A) rpoE knockout does not revert PFLU1583 mutant to first-step motility phenotype. (B) Motility of PFLU1583 Δ48–74 with and without accompanying PFLU1131-del15 mutation. (TIF) [file pbio.3002348.s006.tif]

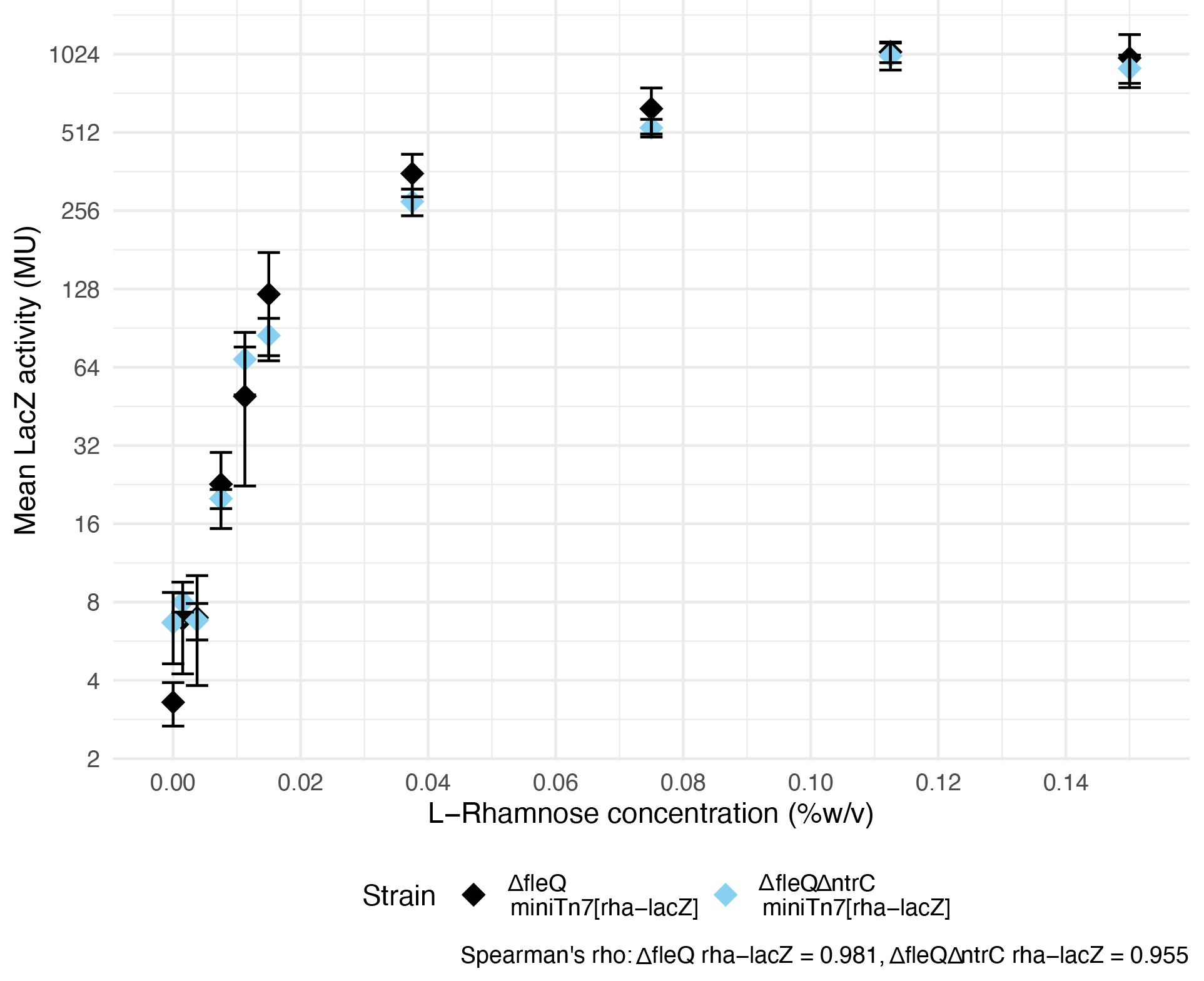

Supplement: S7 Fig — Whiskers represent standard deviation above and below the mean value. Data underlying this figure can be found in S18 File. (TIF) [file pbio.3002348.s007.tif]
