# Supplementary material for: Evolutionary innovation through transcription factor rewiring in microbes is shaped by levels of transcription factor activity, expression, and existing connectivity
Source: PLoS Biol. 2023 Oct 23;21(10):e3002348. doi: 10.1371/journal.pbio.3002348 (PMC10621929; doi:10.1371/journal.pbio.3002348)
Supplement: Attachment — Submitted filename: PLOS B response to reviewers.pdf [file pbio.3002348.s026.pdf]

**Response to Reviewers**

**Reviewer #1:**

**The manuscript of Shepherd et al., describes a study focussed on understanding gene regulatory network rewiring using an elegant experimental model. The authors describe a hierarchy of rewiring in transcription factors to rescue a loss of function mutation. The authors reveal key features for rescuing in GRN - high activation, high expression and low level affinities for target genes. These features reveal that effects can be targeted and global in terms of functional changes with pre-existing architecture of the GRN exerting influences over gain of function. This work has profound implications in terms of our understanding of evolvability of gene networks through TF innovation.**

**Overall the manuscript is beautifully written with a clear narrative.**

**There are a few minor points for the authors to clarify/modify, but otherwise the manuscript makes a significant contribution to understanding in this area and I congratulate the authors on a great piece of work.**

We would like to thank the reviewer for their summary of our work and their kind remarks.

**Minor points**

**Line 83/84 - for non-specialists adding a few words about this strong selection for swimming and perhaps a reference could be useful.**

We have added this – (Lines 86-89)

**Line 100 - would the sentence sound clearer if 'is the only reason' is replaced with 'Perhaps..'**

We have made this change. (Line 114)

**Line 472-473 - I feel this is maybe underplayed a little, and perhaps the authors should make the distinction clearer that in two component systems rather than one-component systems (for want of a better phrase) for example metabolic responsive TFs - Ara, GntR, lac, Tet etc...this is explained a little in the paragraph starting line 495...interestingly, negatively autoregulatory TF maybe have smaller regulons? So perhaps loss of function mutations are more common? To diversify function in two component systems, the way to do this**

**could actually to for heterologous RRs - see** https://eur01.safelinks.protection.outlook.com/?url=https%3A%2F%2Fwww.ncbi.nlm.nih.gov%2Fpmc%2Farticles%2FPMC4125116%2F&data=05%7C01%7Ctt515%40bath.ac.uk%7C7480c2c3461249f9973208db98026e4f%7C377e3d224ea1422db0ad8fcc89406b9e%7C0%7C0%7C638270908431265730%7CUnknown%7CTWFpbGZsb3d8eyJWIjoiMC4wLjAwMDAiLCJQIjoiV2luMzIiLCJBTiI6Ik1haWwiLCJXVCI6Mn0%3D%7C3000%7C%7C%7C&sdata=1tqBWVlriTCU1OlZcn7qaXzMO7ViPU38rHXR6ELNLPs%3D&reserved=0 [ncbi.nlm.nih.gov]

We thank the reviewer for this useful suggestion, and have included additional detail to this section on one-component vs two-component systems (Line 494-496)

**Reviewer #2:**

**This manuscript demonstrates how the loss of a specific regulatory gene can be compensated by secondary mutations that result in rewiring of gene regulatory networks and (partly) restore fitness.**

**The key idea of this study is not novel - several previous studies (several of which are duly cited) have already revealed that 1) evolution is often surprisingly repeatable and 2) that promiscuity greatly facilitates evolutionary adaptation.  However, what sets this manuscript apart from much of the previous body of work is that it uses de novo adaptation (adaptive laboratory evolution) to investigate which transcription factors and regulatory modules in are most likely to drive cellular adaptation to a specific new condition.  As is the case in many other studies, they find that evolutionary adaptation consistently and repeatedly follows a similar mutational trajectory.  Importantly, they go on to study whether other adaptive pathways exist if the most preferred evolutionary route is closed off (by deleting the key transcription factor that is always mutated).  They find that indeed, other pathways do exist, even if these are never chosen if the more preferred adaptive route exists, likely partly because the mutational target size or rate to obtain these alternative suppressor mutants is lower, and partly because they also suffer a fitness deficit compared to the mutants that follow the preferred mutational path.**

**As such, this study contributes to our general understanding of evolution by demonstrating a certain hierarchy or preference in the exact genomic modules (transcription factors) that drive evolutionary adaptation, and shows that the hierarchy is a result of both mutation supply and differences in fitness of the (intermediary) mutants. In addition, the study also confirms that promiscuity is**

**a prime driver of evolutionary adaptation (although I think that this particular conclusion is not only not very novel, but also not very convincing because promiscuity is not directly demonstrated - see major comment below).**

We would like to thank the reviewer for their neat summary of our work and its primary findings.

**Major comments**

**My only major concern regarding this study is that the authors do not provide much experimental evidence for the molecular mechanism by which compensatory mutations work. Instead, they seem to assume that every compensatory mutation always increases "promiscuity"...**

**Throughout the text, the authors use the word promiscuous to denote promiscuous binding of transcription factors or kinase substrate specificity (established terminology) as well as a more general term that seems to refer to alternative regulation that may be due to rewired transcriptional networks or kinase specificity, but for which this has not been demonstrated directly. Instead, the authors seem to assume that the interactions become promiscuous, without providing evidence (eg in line 131, line 240, the title in 248, line 321, the whole paragraph starting at line 326, and line 402...). Unless I am mistaken, promiscuous binding of the transcription factors or kinases was not demonstrated, yet the authors seem to assume that this must be happening. I think it is not OK to call a protein or mutation "promiscuous" without directly showing that it changes the specificity of its activity or interaction. Instead, I would suggest to clearly separate the concept of a promiscuous interaction (between molecules) from the broader concept of changes in physiology and regulation that can be due to multiple mechanisms. May be better to call the latter "transcriptional rewiring" (an established term that the authors also use in the introduction)?**

**The finding that increased expression of transcription factors may stimulate transcriptional rewiring is interesting and plausible. However, again, the authors do not directly show that this is indeed the case (by showing that the over-expressed transcription factor indeed shows more promiscuous binding).**

Our suggestion that the transcription factors may be acting more promiscuously comes from the data that shows increased expression across many other RpoN-EBP controlled genes. However, we agree with the reviewer that our use of the term ‘promiscuous’ is not sufficiently evidenced (via binding assays), and have reworked the manuscript to switch to the more broad term ‘rewiring’, as suggested by the

reviewer. Unpicking the changes in protein-DNA interactions in the evolved motility mutants is an exciting future area of research we are actively pursuing.

**Minor**

**The "Results" section starts a bit abruptly, without sufficient introduction. I think it is useful to inform the reader (again) about the outcome of previous experiments before asking the questions what the reason for this outcome (the repeated mutations in NtrC) could be...**

We have added a sentence to reinform the reader of previous experimental outcomes (Lines 112 -113)

**Line 114: what is a "sensor histidine kinase"? Please explain in text.**

We have added explanation to clarify the use of this term (Lines 129-131)

**It might be personal preference, but I am not a fan of naming the two adaptive evolutionary paths the "commonly rewired route" and the "unmasked rewiring pathway". What about "primary adaptive route" and "secondary adaptive route", or a name that alludes to suppression?**

We have changed the names for these pathways to “primary” and “alternative”. We avoid use of “secondary”, as we already refer to secondary and second-step mutations, which can become confusing when the same word is used to refer to two separate things.

**Line 237: typo - "severE"**

This has been corrected.

**Line 280 : act on RpoN, the partner sigma factor of PFLU1132, (add comma's)**

This has been corrected

**Discussion section: It would be interesting to compare the results with that of a recent very similar study published in Molecular Biology and Evolution, where Helsen and coworkers perform a very similar series of experiments by evolving more than 200 parallel populations as they adapt to the loss of a specific gene (with multiple gene deletions being tested). Here too, the authors find reproducible adaptive mutational paths as well as primary and secondary mutations, with the latter ones restoring fitness. Moreover, they find that compensation for the loss of highly connected "hub" genes generates more in adaptive paths, as well as the fitness that the adapted mutants reach (reference:**

**PMC7530610)**

We thank the reviewer for suggesting this interesting work, which we now mention in the discussion (Lines 520-526).

**Reviewer #3:**

**This manuscript is a follow-up project from a study published in 2015. In that initial work, the transcription factor FleQ controlling flagellar synthesis in Pseudomonas fluorescence was deleted and the evolutionary rescue of the motility was studied. In all replicates, a homologous transcription factor, NtrC, was recruited to control the flagellar genes.**

**Here, the same evolutionary experiment was performed, but this time in a strain that has FleQ and NtrC deleted. This time, another homologous transcription factor, namely PFLU1132, was recruited to control the flagellar genes. Mutations are identified through genome sequencing and gene expression levels are monitored by RNA sequencing. The authors try then to draw general conclusions about what factors favor the recruitment of a homologous transcription factor and why this happens faster/more frequently for NtrC than for PFLU1132. They conclude that for NtrC provides a better and easier reached solution. They also conclude that increasing activation and expression of the recruited transcription factors helps to increase the off-target activity.**

**This manuscript addresses an important question. It presents a nice system to study this question and the findings are interesting. The study could have been even stronger, if combined with some biochemical analysis to confirm putative mechanisms, such as phosphorylation levels or confirmation of putative binding sites or putative increased readthrough.**
**Nevertheless, I support publication if my comments are addressed.**

We would like to thank the reviewer for their summary of our work and its conclusions, and for their kind words about our research. We feel that the novelty of this research approach is that it uses experimental evolution to determine the evolutionary accessibility of different mutational pathways leading to transcription factor innovation, and in doing so reveals constraining factors. This study leaves exciting open questions to many future avenues of research that we are actively pursuing, such as DNA-protein interactions and phosphorylation activity. However, we feel that this is beyond the scope of this work. We would also like to bring a recent paper to the reviewer's attention: Ghose et al 2023 (PNAS) [PMID: 37098061]. Where they use fluorescent expression analysis on mutant libraries (Sort-seq) to

quantify crosstalk (or cross-phosphorylation) between a paralogous family of bacterial signalling proteins. This paper highlights the use of expression analysis to determine interactions between paralogous proteins driven by few mutational differences, without invoking biochemical analysis.

**My comments mainly aim to increase clarity and understandability, as well as tuning down some claims.**

**Major comments:**

* **The manuscript talks many times about "gaining promiscuity". For me, changing promiscuity is changing the binding affinity of the transcription factor to the DNA by either mutating the protein or the DNA binding site. However, if I understood correctly, neither of them happens here (in contrast to the initial study where the transcription factor NtrC acquires a mutation in the DNA binding site). Here, it simply increases off-target binding by increasing the expression levels of PFLU1132 and its activity by phosphorylation via PFLU1131. I think this should be re-formulated throughout the manuscript to make clearer.**

We have now modified the manuscript to remove reference to promiscuity. We instead refer to rewiring (gain of new regulatory connections), which our results support.

* **Even though it is likely that the mutation in PFLU1131 increases phosphorylation of PFLU1132 and therefore its activity, the manuscript does not present any direct proof, e.g. by western blot analysis for the phosphorylated protein. I am not saying the authors have absolutely to do this, but I think the claims have to be tuned down.**

We thank the reviewer for pointing this out. We have changed the text so as not to suggest speculation on whether there is increased phosphorylation of PFLU1132. (Line 285)

* **Lines 166-174 is a repetition of the previous section. I found this very confusing. Find a better way to make the connection between the two sections.**

We thank the reviewer for highlighting this. This section has been reworded to remove repetitive portions and clarify the purpose of the experiment and result being outlined. (Lines 178-182)

* **Often, the background in which a mutation was tested is not clearly**

**indicated. E.g. Fig. 4BC. I believe PFLU1131-del15 is in a ΔfleQΔntrC background, but it is not mentioned. The same for Figure 5CD**

The reviewer is correct, the PFLU1131-del15 mutation is in a ΔfleQΔntrC background. Clear statements have been added to the legends of these figures to clarify this.

***	In Fig. 2B it is not at all clear which picture corresponds to which strain tested. In your preprint, the pictures are labelled (but also unclear, as the background is missing, see comment above), but in this version, I don't see any labels at all?!? Maybe make schematic drawings to make this clearer and easier to understand?**

The authors are unsure why the labels are missing from the version provided to the reviewers and apologise for this issue. We have added labels and added a note in the legend for this figure section noting the genetic background for all strains tested.

**Minor comments:**

*	**Separating figures from and captions from the main text makes the life of reviewers very difficult. For next time (especially for a first round), please embed the figures.**

We are following PloS Biology Author Submission guidelines, where is says, "Upload separately. Do not put figure files in the manuscript body…." And for Figure captions, "Insert immediately in the manuscript text after the first paragraph in which the figure is cited". As such, we will leave these as requested in the submission guidelines. We can recommend though that the reviewer reorder pages of the pdf to move figures closer to the figure caption.

***	In the beginning, please explain in few sentences why there is a strong selection for motility (local nutrients become depleted). It is not very clearly explained. It only become clear to me when reading the initial study.**

This clarification has now been added (Lines 86-89)

***	In the introduction and/or discussion, discuss the importance of gene duplication. Those transcription factors are homologs, i.e. generated by gene duplication. However, I did not read gene duplication a single time in the manuscript. I think this should be at least shortly elaborated.**

Mention of gene duplication and its role in facilitating opportunities for rewiring has been added to the introduction (Line 63-65) and the discussion (Line 436-439).

***     In the discussion, I would also appreciate a comment about alternative mechanisms that could lead to rewiring, e.g. by mutation of the cis-regulatory elements.**

This has been added to the discussion (Line 523-531)

***     Line 179 refers to Supporting Fig. S3, but this figure shows growth curves related to Fig. 4. It should probably say Fig. S4.**

This has been amended.

***     For the first time use (line 124), write SNP out**

This has been added.

*     **Line 89: "additional transcription factors" it is only one (PFLU1132), no?**

We have amended this.

***     Line 118: quickly explain a two-component system. Not everybody will be familiar with it.**

A clarification has been added (Line 136-137).

***     After Supporting Figure S2 you cite Supporting Figure S4. Usually, they should named in order of appearance.**

This has been corrected.

***     Line 183 cites ref. 34, but I think it should be ref. 31**

This was indeed an error, it has been corrected.

***     Figure caption 3: "ΔfleQΔntrC first step" is a strange expression. I get what you mean, but it would be more precise to write something like "strain with mutations acquired in the first step, i.e. ΔfleQΔntrC PFLU331-del15"**

This has been amended

***     ΔfleQ ntrB-T97P appears the first time in Figure 3 and then Figure 4. It needs some explanation in the main text.**

This has been added to the text (line 202)

***	Fig. 4A. Can you do statistical tests comparing the two 1st steps together and another comparing the two 2nd steps together? That would seem more relevant**

This has been added to Figure 4A. Faceting has been removed to facilitate addition of these comparisons as significance brackets.

***	Line 200. I think instead of "considered" measured or compared would be more appropriate**

This has been amended to measured

***	Line 243: For the cells having a low pleiotropic fitness cost is in principle a good thing, so that per se does not make it a poorer solution. It is just an indication that the expression level is lower**

We have amended this paragraph to reemphasis that the solution is poorer in terms of flagellar motility specifically (Lines 255-261)

***	Fig. 6, maybe also indicate the putative positive feedback loop. It took me a while to understand RpoN binding site would mean that PFLU1132 might activate transcription there.**

An arrow indicating a putative positive feedback loop has been added to Fig. 6A.

***	Line 470: It is not only the topology of the GRN (e.g. negative vs positive feedback), but also the local sequence context that matters. For example, the increase in read-through by deleting a terminator is not really connected with the network topology (architecture), but the local sequence context.**

We have included reference to local sequence context here (Line 491-492).

***	Supporting Fig. S3: Why do some growth curves start below 0? Did you also try other measures to analyse these curves, such as growth rates? Are your conclusions robust to the measure you choose?**

This was a plotting error which has been corrected.

The curves presented in Fig. S3 are the raw data used to calculate the area under growth curve (AUGC) metrics presented in Fig. 4C. Area under the growth curve is a robust measure of overall growth fitness, accounting for variance in the lag phase

duration, max growth rate, and carrying capacity of the growth cycle. We provide the raw curves in the supplementary material so that readers can inspect these, as it is possible for similar AUGC values to be produced by curves of different shapes (the raw curves allow us to validate this) – See Sprouffske et al., 2016 for further details on growth curve analysis - http://dx.doi.org/10.1186/s12859-016-1016-7

***	Supporting Fig. S6. I believe A and B are inversed (either in the caption or in the figure, but the two don't fit together).**

This is correct and has been amended. Apologies for this oversight.

**Reviewer #4:**

**The authors present a study on gene regulatory network "rewiring" (i.e. a change in GRN connections) mediated by transcription factor promiscuity in the bacterium Pseudomonas fluorescens. They find that there is a hierarchy among TFs that are rewired to rescue lost function if the primary pathway is eliminated and identify three key properties (high activation, high expression, and low-level affinity for novel targets) that determine TF factor evolvability. While gene network evolution is an interesting, timely, and important topic, my concerns below must be addressed before I can support publications in PLoS Biology.**

We thank the reviewer for their summary of our work, and kind words about our research.

**Major Concerns:**

**-I struggle to see the biological/evolutionary relevance of this study. I am not clear of why GRN re-wiring is relevant to the experimental model system considered. If there was a fitness disadvantage in terms of Pseudomonas fluorescens' ability to swim for all rewired networks, under which scenarios would these alternative networks evolve? Even if a rewiring mutation occurred in a small number of cells in population it would be selected against and disappear from a microbial population unless there was a fitness advantage or in a regime where genetic drift applies. Also, what is the motivation for investigating the evolutionary rescue of a non-RpoN dependent trait? Finally, the authors should discuss the limitations of their study; specifically that their findings are specific to the GRNs with transcription factors homologous to the target transcription factor.**

Our study used a model system to understand fundamental evolutionary principles and factors influencing transcription factor evolution through rewiring. Utilising the rescue of motility works well as a model phenotype, as it is easily observable and easy to select for. We would not expect the specific alternative networks that evolve in our laboratory experiment to evolve in nature. The evolutionary processes we document serve as a representation of such processes which may occur in nature (evolution by gene loss is common in many bacteria (Bolotin and Hershberg, 2016) and often evolve rapidly upon encountering novel environments (Ali and Seshasayee, 2020)).

Flagellar motility is an RpoN dependent trait in our study organism, Pseudomonas fluorescens SBW25, hence our focus on this.

We have added discussion of the limitation suggested by the reviewer to the manuscript (Line 446 - 448)

**-If high expression of the transcription factors (PFLU1132 or ntrC) lead to promiscuity, how do you know promiscuity is not facilitated due to the expression of these genes, rather than promiscuity being facilitated by the saturation of the cognate binding sites? Also, are you sure that the GRNs are being re-wired as opposed to independent/compensatory mutations arising to restore the slow and fast swimming phenotypes?**

It is possible that saturation of cognate binding sites is occurring, which aligns with our findings as such an effect would be dependent on high transcription factor expression. We have not directly tested this, so cannot conclude on a mechanism in this manuscript – this is an exciting research question that we plan to investigate in the future.

Our engineered strains contain a complete FleQ knock-out. The only way for flagellar genes to be expressed in the absence of FleQ, is for something else to bind to FleQ target sites and initiation transcription. Whole genome sequencing revealed the mutations in our evolved motile isolates are outside of the flagellar network. Only rewiring (i.e., recruitment of a non-cognate transcription factor to initiate flagellar motility), could recover swimming motility.

**-The results of this study are not properly put into context of previous work. 1) There are several relevant publications from Mads Kaern and Gabor Balazsi's groups on how GRN motifs affect drug resistance evolution (see Charlebois et al., Phys. Rev. E, 2014; Gonzalez et al., Mol. Syst. Biol., 2015; Farquhar et al., Nat. Commun., 2019; Camellato et al., Eng. Biol., 2019) that should be incorporated into the manuscript;**

We have incorporated reference to these works in the introduction (Line 49)

**2) The authors have previously published a study on PFLU1131 (https://eur01.safelinks.protection.outlook.com/?url=https%3A%2F%2Fdoi.org%2F10.1093%2Fmolbev%2Fmsac132&data=05%7C01%7Ctt515%40bath.ac.uk%7C7480c2c3461249f9973208db98026e4f%7C377e3d224ea1422db0ad8fcc89406b9e%7C0%7C0%7C638270908431265730%7CUnknown%7CTWFpbGZsb3d8eyJWIjoiMC4wLjAwMDAiLCJQIjoiV2luMzIiLCJBTiI6Ik1haWwiLCJXVCI6Mn0%3D%7C3000%7C%7C%7C&sdata=DfI%2BsMIpMGfQ%2FpoyHFMmEdhLf%2Fplm%2FZa4H79F3cxXCg%3D&reserved=0 [doi.org]), which they should discuss when they state that PFLU1131 remains unstudied;**

We have added this when mentioning the lack of research on PFLU1131 (Line 128-129)

**3) MacLean et al., PNAS, 2004 should be cited when discussing pleiotropic fitness cost and central carbon metabolism.**

This has been added (Line 316)

**Minor Concerns:**

**Why was there no PFLU1131 knockout created? Perhaps PFLU1132 could have worked with another component?**

The purpose of the PFLU1132 knockout was to demonstrate that the observed PFLU1131 mutations rescue motility through the transcription factor PFLU1132, and not through another regulator. This was important for our study, as it allowed us to focus subsequent investigations and conclusions on the PFLU1132 transcription factor. A PFLU1131 knockout investigates a separate, but interesting research question, around whether PFLU1132 can act with any other partners for rewiring, which we would like to investigate in the future.

**It is stated multiple times that the pathway of ntrBC is always utilized even in the presence of the other PFLU1131/2 pathway, yet in line 166, it is stated that the first step PFLU1131 mutation would lead to motility even if ntrC is present?**

This is correct. This was an important control for our study, as it demonstrates that the observed rescue of motility through PFLU1131 mutations does not depend on the absence of ntrC (the genetic background they evolved within) – the pathways are not mutually exclusive. If this was the case, the explanation for why NtrC is rewired in preference to PFLU1132 would be simple – PFLU1132 cannot rewire when NtrC is

present. This test demonstrates that PFLU1131 mutations grant motility in the presence of NtrC, so we can be sure that the observed hierarchy between the two pathways is not due to such mechanistic dependencies, but is instead an evolutionary one, likely based on both the speed at which ntrB mutates (Horton et al., 2021, Shepherd et al., 2022), and the poor motility phenotype provided by PFLU1131 (Fig. 4), which we elaborate upon further.

We have added to this part of the text, to clarify the significancy of this test to readers (Line 163-166).

**Introduction, first paragraph: The homologous transcription factor and its relation to the FleQ and ntrC is not clear. Mention specifically on how they are structurally related, and how the level of homology is determined.**

We believe the reviewer is in fact referring to the first paragraph of the results section, not the introduction, as this is where the relevant information is given in detail. We have amended the text of this section to include this (Lines 131 -137).

**Line 67: Provide a biological example of transcription factor promiscuity to motive the study, in addition to the general statement/references provided.**

This has been added (Lines 76-79)

**-Section on "Motility evolution experiments": How exactly is motility determined and how is this differentiated from colony expansion due to cell division? The authors provide references to previous work, but it would be helpful if these details were explicitly included in the manuscript.**

The methods section has been updated to clarify this, and provide supporting references (Lines 596-600)

In our study system, flagellar motility is visually distinct from all other forms of motility in *P. fluorescens*, being characterised as a zone of growth, shaped in a semicircle, which is present through the entire depth of the agar plate. Movement by simple colony expansion occurs only on the surface of the soft agar, not throughout its depth. Motile isolates were identified through visual inspection for this phenotype.

**Figure 2: Caption needs to be improved to include more details. I am not clear what each panel B is supposed to show? What do black data points denote? Same comment for Fig. 5C,D.**

These figure legends have been clarified.

**-Figure 3 caption, second sentence: Should read "ΔfleQΔntrC and ΔfleQΔntrC del" and not "ΔfleQΔntrC and ΔfleQΔntrC"?**

This has been corrected and clarified.

**Style & Grammar**

**-Use quotations the first time you use words like "circuits", "wires", "crosstalk" etc. in a biological context.**

This is a stylistic choice that is not common or consistently used in the literature, and the authors prefer not to add undue emphasis in a way that might reduce clarity for the reader.

**-Line 363: "PFLU1131/2"  not "PFLU113/2".**

We would like to thank the reviewer for highlighting this typo. This has been corrected.

**-Line 462: Define CF.**

As we only mention cystic fibrosis (CF) once in the text, the acronym has been switched to the full term

**-Line 501: "enable innovation"?**

This has been amended

**-Discussion: Remove bolded text.**

This is a stylistic choice by the authors to add emphasis and clarity to the discussion section, and we feel clarity will be reduced through its removal.

**-To enhance clarity, I suggest that the paragraphs explaining the two step mutations should come before all the other paragraphs discussing first or second step mutations.**

The authors are unsure which section of the manuscript the reviewer is referring to. Two-step mutations are already discussed at the beginning the results section. The discussion section does not feature a paragraph about the observed two-step process.

**-Figure captions: Be consistent with formatting and clearly state the intended message of each figure in the figure title/first sentence of the caption.**

Figure caption titles have been amended as requested.
