# Supplementary material for: Evolutionary innovation through transcription factor rewiring in microbes is shaped by levels of transcription factor activity, expression, and existing connectivity
Source: PLoS Biol. 2023 Oct 23;21(10):e3002348. doi: 10.1371/journal.pbio.3002348 (PMC10621929; doi:10.1371/journal.pbio.3002348)
Supplement: Attachment — Submitted filename: PLOS B response to reviewers_v3.docx [file pbio.3002348.s027.docx]

**Response to Reviewers**

**Academic Editor comments:**

Based on our Academic Editor's assessment of your revision, we are likely to accept this manuscript for publication, provided you satisfactorily address the following data and other policy-related requests.

a) We note that the Title seems somewhat self-evident, and we wonder whether you would be able to modify it to more explicitly include some ideas of which attributes of pre-existing GRN architecture are important (i.e. the three "key properties" mentioned in the Abstract)?

**We have changed this title in line your suggestion to “Evolutionary innovation through transcription factor rewiring in microbes is shaped by levels of transcription factor activity, expression and existing connectivity”**

b) Please include the study species in the Abstract.

**We now refer to the study species used in lines 28-29**

c) Please address my Data Policy requests below; specifically, we need you to supply the numerical values underlying Figs 2C, 3, 4ABC, 5BCDE, 6B, 7, S3, S4, S5, S7, either as a supplementary data file or as a permanent DOI’d deposition. I note that you already have an associated OSF deposition, so you could add the underlying data to those files (some of which may already contain data relating to the Figs) or provide extra supplementary files.

**We now supply all raw data underlying figures. This is provided both as a supplementary files and is available in the associated OSF deposition.**

d) Please cite the location of the data clearly in all relevant main and supplementary Figure legends, e.g. “The data underlying this Figure can be found in S1 Data” or “The data underlying this Figure can be found in [https://eur01.safelinks.protection.outlook.com/?url=https%3A%2F%2Fdoi.org%2FXXXXX&data=05%7C01%7Ctt515%40bath.ac.uk%7Cd040e6e9a69c4f059c1d08dbb511331a%7C377e3d224ea1422db0ad8fcc89406b9e%7C0%7C0%7C638302857696131321%7CUnknown%7CTWFpbGZsb3d8eyJWIjoiMC4wLjAwMDAiLCJQIjoiV2luMzIiLCJBTiI6Ik1haWwiLCJXVCI6Mn0%3D%7C3000%7C%7C%7C&sdata=wbcXFirpfQP5ns3mWn4%2BaLq7S%2FkH3HtDUyWfd0jipHs%3D&reserved=0"](https://eur01.safelinks.protection.outlook.com/?url=https%3A%2F%2Fdoi.org%2FXXXXX&data=05%7C01%7Ctt515%40bath.ac.uk%7Cd040e6e9a69c4f059c1d08dbb511331a%7C377e3d224ea1422db0ad8fcc89406b9e%7C0%7C0%7C638302857696131321%7CUnknown%7CTWFpbGZsb3d8eyJWIjoiMC4wLjAwMDAiLCJQIjoiV2luMzIiLCJBTiI6Ik1haWwiLCJXVCI6Mn0%3D%7C3000%7C%7C%7C&sdata=wbcXFirpfQP5ns3mWn4%2BaLq7S%2FkH3HtDUyWfd0jipHs%3D&reserved=0%22)

**This has been included to all figure legends.**

e) Please make any custom code available, either as a supplementary file or as part of your OSF deposition.

**No custom code was used. Where R packages have been used to generate plots or statistical test these have been cited in the text.**
